# Genome-wide subcellular localization of putative outer membrane and extracellular proteins in *Leptospira interrogans *serovar Lai genome using bioinformatics approaches

**DOI:** 10.1186/1471-2164-9-181

**Published:** 2008-04-21

**Authors:** Wasna Viratyosin, Supawadee Ingsriswang, Eakasit Pacharawongsakda, Prasit Palittapongarnpim

**Affiliations:** 1 BIOTEC Central Research Unit, National Center for Genetic Engineering and Biotechnology, Pathumthani, 12120, Thailand; 2 Department of Microbiology, Faculty of Science, Mahidol University, Bangkok, 10400, Thailand

## Abstract

**Background:**

In bacterial pathogens, both cell surface-exposed outer membrane proteins and proteins secreted into the extracellular environment play crucial roles in host-pathogen interaction and pathogenesis. Considerable efforts have been made to identify outer membrane (OM) and extracellular (EX) proteins produced by *Leptospira interrogans*, which may be used as novel targets for the development of infection markers and leptospirosis vaccines.

**Result:**

In this study we used a novel computational framework based on combined prediction methods with deduction concept to identify putative OM and EX proteins encoded by the *Leptospira interrogans *genome. The framework consists of the following steps: (1) identifying proteins homologous to known proteins in subcellular localization databases derived from the "consensus vote" of computational predictions, (2) incorporating homology based search and structural information to enhance gene annotation and functional identification to infer the specific structural characters and localizations, and (3) developing a specific classifier for cytoplasmic proteins (CP) and cytoplasmic membrane proteins (CM) using Linear discriminant analysis (LDA). We have identified 114 putative EX and 63 putative OM proteins, of which 41% are conserved or hypothetical proteins containing sequence and/or protein folding structures similar to those of known EX and OM proteins.

**Conclusion:**

Overall results derived from the combined computational analysis correlate with the available experimental evidence. This is the most extensive *in silico *protein subcellular localization identification to date for *Leptospira interrogans *serovar Lai genome that may be useful in protein annotation, discovery of novel genes and understanding the biology of Leptospira.

## Background

Leptospirosis is a globally widespread zoonosis caused by the animal spirochete pathogen *Leptospira interrogans *[1]. The clinical feature of its severe disease form, known as Weil's syndrome, or acute renal failure, is associated with multiple system complications, including renal failure, meningitis, and pulmonary haemorrhage. Although early treatment for leptospirosis is important for ensuring a favorable clinical outcome, this is often difficult to achieve, as symptoms during the early stages of infection resemble those of several other systematic diseases.

One potential method for controlling the spread of leptospirosis is through the development of vaccines. Candidates for vaccine production include outer membrane (OM) and extracellular (EX) proteins, several of which have been implicated in chemotaxis, adherence and other pathogenic steps. Attempts to identify such proteins have been performed previously by experimental [2-14] and computational methods [15-20]. Complete genome sequences of two serovars, Lai and Copenhageni of *L. interrogans *have been reported [15-17]. Hundreds of putative membrane proteins and lipoproteins were predicted, although in many cases, gene annotation may be incomplete or inaccurate to reliably identify putative vaccine candidates.

Previous studies have tried to identify potential vaccine candidates using experimental methods and *in silico *predictions. Proteomic analysis of purified outer membrane vesicles (OMVs) of *L. interrogans *serovar Copenhageni was performed by Nally *et al*. and revealed 33 intact OM proteins [13]. The study by Gamberini *et al*. [18] showed 16 predicted surface exposed lipoproteins of *L. interrogans *serovar Copenhageni via whole genome analysis, only four of which are conserved among 8 pathogenic serovars. Since leptospiral lipoproteins are usually (but not exclusively) surface exposed proteins, and many are vaccine candidates, Setubal *et al*. [19] focused on lipoprotein prediction using spirochaetal lipoprotein (SpLip) program and identified 146 predicted lipoproteins (but not their localizations) for *L. interrogans *serovar Lai. The search for new potential vaccine candidates was continued by Yang *et al*. [20], who used a filtering approach combining in *silico *analysis, comparative genome hybridization, and microarray methods to identify 226 leptospiral surface exposed proteins. All of the previous studies summarized above focus on identification of vaccine candidates.

However, both computational and experimental have their own drawbacks [21,22] Computational methods, for instance, depend on the presence of type I signal peptides [23,24], transmembrane helices [24-26], or other particular features specifically found in previously identified membrane proteins, which may not be highly specific or sensitive. Experimental methods, on the other hand, yield results that may be complicated by cross-compartment contamination occurring during the preparation of samples, which can also result in the inclusion of false positive results in data sets [21,22]. Hence, results obtained from both methods can occasionally lead to conflicting conclusions. We believe that such a focused approach without attempt to accurately identify periplasmic proteins (PP) and cytoplasmic membrane (CM) proteins can lead to erroneous identification of PP and CM as OM or EX by both *in silico *and experimental approaches. A holistic prediction of all membrane protein localizations will lead to better accuracy in genome annotation of membrane proteins, including vaccine candidates.

In this study we utilized a combination of three computational prediction tools PSORTb [27,28], Proteome Analyst (PA) [29], and ProtCompB [30] to perform whole genome analysis of protein subcellular localization, and to identify novel putative *L. interrogans *serovar Lai OM and EX vaccine candidates. We combined the results derived from these three prediction algorithms into a consensus vote, resulting in a more accurate protein subcellular localization prediction. Furthermore, we incorporated homology searching against the DBSubloc database [31] and structural information from the GTD prediction [32] to enhance genome annotation, and to infer OM, EX and PP localized proteins. We also developed a specific classifier based on Linear Discriminant Analysis (LDA) for identification of leptospiral cytoplasmic proteins (CP) and cytoplasmic membrane proteins (CM), using a training set obtained from the consensus vote. We were able to assign subcellular localizations to several previously uncharacterized hypothetical proteins, thus improving *L interrogans *genome annotation.

## Results

**Figure 1:**
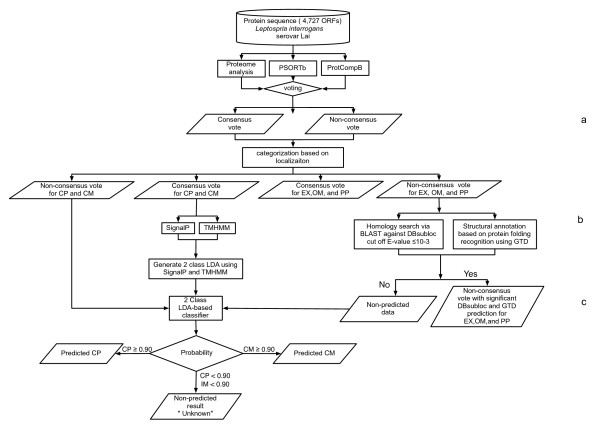
**Flow chart of the method used for subcellular localizations of *Leptospira interrogans *serovar Lai genome**. Protein sequences of *Leptospira interrogans *serovar Lai genome (4,727 ORFs) were analyzed for subcellular localization using PSORTb, ProtCompB, and Proteome analyst (PA) prediction. (a) The consensus vote was obtained from the majority vote type procedure to obtain the result with high prediction accuracy. If all 3 methods agree for localization it was assigned as a consensus vote. The remaining (1 or 2 out of 3 predicted result) was assigned as non-consensus vote. The consensus vote of CP and CM was used as a training set for the development of an LDA-based classifier for CP and CM in the next step. (b) The non-consensus vote results of OM, PP, and EX were further analyzed for sequence and structure homology by DBsubloc and GTD prediction. The non-consensus vote of EX, OM, and PP with significant homology or/and structure information were identified by DBsubloc and GTD prediction. (c) Non-consensus votes of CP, CM and the non predicted data from DBsubloc and GTD predictions were further analyzed for subcellular localization using LDA-based classifier for CP and CM. Significantly predicted results were proteins classified with more than 0.90 probability for CP and CM proteins. The remaining queries that could not be identified in this step were classified as "unknown" results.

We performed the subcellular localization prediction of *L. interrogans *serovar Lai using the pipeline described in the Material and methods section (shown in Figure 1), following the steps of training set verification, consensus vote, homology and structural prediction, and finally LDA-based classification.

### Training set verification: Localization predictions of a set of experimentally verified proteins with known localization

To evaluate the robustness and versatility of our protein localization procedure, we used a set of well- characterized Gram-negative bacterial proteins with experimentally verified localizations taken from the work by Gardy and Brinkman [22] as a test set. The data set comprising 299 proteins was first analyzed by using PSORTb, PA, and ProtCompB. We found that, individually, PSORTb, PA, and ProtCompB assigned 73%, 71% and 79% of the verified protein localizations respectively (recall rate in Table 1). The overall precision rates were 97%, 95 and 83%, respectively. As expected, the overall recall rate was highest for ProtCompB, while its precision rate was also the lowest. The recall rate based on "consensus vote" (see materials and methods) results derived from all three methods was 48% without any false positives. Relaxing the criteria by considering predicted results of any two methods or the "majority vote" resulted in an overall recall rate of 77% with a single false positive.

**Table 1:** Localization predictions of a set of 299 experimentally verified proteins with known localization

Actual localization	Total	TP	FP	FN	TN	Precision	Recall
**PSORTb**							
CP	145	110	1	35	110	99.10%	75.86%
CM	69	55	2	14	166	96.49%	79.71%
PP	29	18	0	11	207	100.00%	62.07%
OM	38	30	0	8	195	100.00%	78.95%
EX	18	6	3	12	216	66.67%	33.33%
Total	299	219	6	80	894	97.33%	73.24%
**Proteome Analyst**							
CP	145	94	0	51	119	100.00%	64.83%
CM	69	59	2	10	162	96.72%	85.51%
PP	29	19	3	10	201	86.36%	65.52%
OM	38	31	0	7	192	100.00%	81.58%
EX	18	9	6	9	207	60.00%	50.00%
Total	299	212	11	87	881	95.07%	70.90%
**ProtCompB**							
CP	145	127	11	18	144	92.03%	87.59%
CM	69	55	7	14	227	88.71%	79.71%
PP	29	19	9	10	261	67.86%	65.52%
OM	38	23	18	15	243	56.10%	60.53%
EX	18	11	4	7	277	73.33%	61.11%
Total	299	235	49	64	1152	82.75%	78.60%
**Consensus vote**							
CP	145	67	0	78	154	100.00%	46.21%
CM	69	43	0	26	230	100.00%	62.32%
PP	29	11	0	18	270	100.00%	37.93%
OM	38	19	0	19	261	100.00%	50.00%
EX	18	4	0	13	216	100.00%	23.53%
Total	299	144	0	154	1131	100.00%	48.32%
**Majority vote (2 out of 3 predictions)**							
CP	145	121	0	24	154	100.00%	83.45%
CM	69	59	0	10	230	100.00%	85.51%
PP	29	17	0	12	270	100.00%	58.62%
OM	38	29	0	10	213	100.00%	74.36%
EX	18	6	1	12	215	85.71%	33.33%
Total	299	232	1	68	1082	99.57%	77.33%
**Combination method**							
CP	N/A	N/A	N/A	N/A	N/A	N/A	N/A
CM	N/A	N/A	N/A	N/A	N/A	N/A	N/A
PP	29	25	0	4	56	100.00%	86.20%
OM	38	34	1	4	46	97.14%	89.47%
EX	18	12	2	6	65	85.71%	66.67%
Total	85	71	3	14	167	95.95%	87.53%

299 proteins obtained from the test set used in comparison study by Gardy and Brinkman [22] Majority vote is the result from 2 out of 3 predictions. Combination method: the result from non-consensus vote with significant DBsubloc [31] and/or GTD prediction [32] Precision is calculated as TP/(TP+FP), Recall is calculated as TP/(TP+FN) TP = true positive, TN = true negative, FP = false positive, FN = false negative, N/A= Not applicable

Since the number of outputs for EX and OM proteins agreed by all three predictions was low (low recall rate), we used structure-based homology information from GTD and/or homology search results from DBSubloc prediction as the additional information for inferring protein localization. Using this information, we assessed the likelihood of the "non-consensus vote" outputs (see material and methods) for being EX or OM proteins. When the information from DBSubloc and GTD predictions were also used, the overall recall rates for the EX, OM and PP increased to 67%, 89% and 86% respectively as shown in Table 1. The method resulted in 96% precision. This performance was much better than any of the three individual methods, or any of the above combinations. Therefore, we have shown that the combination of prediction tools, DBSubloc homology search and GTD structural-based prediction markedly improved the accuracy and recall for EX, OM and PP protein localization prediction. Therefore, our prediction pipeline is applicable for subcellular localization prediction of hypothetical, or unknown proteins.

### Subcellular localization predictions of *L. interrogans*: Step 1 Consensus votes

After demonstration of the accuracy of our pipeline prediction with the training set, the whole predicted proteome of *L. interrogans *serovar Lai was analyzed using three computational predictions for protein subcellular localization: PSORTb, ProtCompB, and Proteome analyst (PA). The results obtained from each prediction program are shown in Table 2. ProtCompB assigned subcellular localizations to all protein queries whereas approximately 50% of protein queries were assigned as unknown localization by PSORTb and PA.

**Table 2:** Predicted protein subcellular localizations of *L. interrogans *by PSORb, PA, ProtCompB and consensus vote predictions.

Localization	Subcellular localization prediction			
	
	PSORTb	PA	ProtCompB	Consensus vote
Cytoplasm (CP)	1125	921	2013	418
Cytoplasmic membrane (CM)	606	715	1726*	332
Outer membrane (OM)	112	28		15
Periplasmic (PP)	30	86	478	17
Extracellular (EX)	29	326	510	15
Unknown	2825	2652	-	3930

* Note that ProtCompB prediction in this version, CM and OM were predicted as membrane proteins.

After inspection of the prediction results derived from the three prediction algorithms, it was found that 797 out of 4,727 ORFs of *L. interrogans *serovar Lai genome had the following consensus vote predicted localizations: 418 cytoplasmic proteins (CP), 332 cytoplasmic membrane proteins (CM), 17 periplasmic proteins (PP), 15 outer membrane proteins (OM), and 15 extracellular/secreted proteins (EX) (Table 2, 3, 4 Additional file 1, 2, 3). The biological functions of most of the localized proteins are already annotated. Only about 9% (68 of 797 ORFs) were proteins annotated as conserved hypothetical or unknown proteins. This shows that the consensus vote approach has a high accuracy of subcellular localization prediction for *L. interrogans*. However, this recall of these methods is unacceptably low, since the localization of the majority of proteins remains unknown (3930 out of 4727 proteins).

**Table 3:** Putative extracellular proteins (EX) predicted by the consensus vote

**Lai Locus**	**Copen Locus**	**Protein annotation**
LA3731	LIC10497	Fmh-like protein/hypothetical protein
LA0587	LIC12988	Lactonizing lipase/lipase
LA0872	LIC12760	Microbial collagenase
LA1450	LIC12302	Probable O-sialoglycoprotein endopeptidase
LA2448	LIC10830	Putative outermembrane protein/putative lipoprotein
LA1765	LIC12047	Rhs family protein/cytoplasmic membrane protein
LA4161	LIC13320	Thermolysin/thermolysin precursor
LA4164	LIC13321	Thermolysin/thermolysin homolog precursor
LA2303	LIC11634	3-oxoacyl- [acyl-carrier protein] reductase/CsgA
LA0873	LIC12759	LRR containing protein/cytoplasmic membrane protein
LA2964	LIC11098	LRR containing protein/conserved hypothetical protein
LA3028	LIC11051	LRR containing protein/conserved hypothetical protein
LA3320	LIC10831	LRR containing protein/conserved hypothetical protein
LA3323	LIC10829	LRR containing protein/conserved hypothetical protein
LA0709	LIC12896	Unknown protein/conserved hypothetical protein

Note LRR: Leucine-rich repeat

**Table 4:** Putative outer membrane proteins (OM) predicted by the consensus vote

**Lai Locus**	**Copen Locus**	**Protein annotation**
LA2375	LIC11570	General secretory pathway protein D
LA3149	LIC10964	Hemin receptor/TonB-dependent outer membrane hemin receptor
LB328	LIC20250	Outer membrane protein OmpA/PG-associated CM protein
LA3615	LIC10592	Outer membrane protein OmpA family/PG-associated CM protein
LA1963	LIC11941	Outer membrane protein precursor CzcC/heavy metal efflux pump
LA3927	LIC13135	Outer membrane protein tolC precursor/outer membrane protein
LA1356	LIC12374	Probable TonB-dependent receptor
LA2641	LIC11345	Probable TonB-dependent receptor/ferrichrome-iron receptor
LA3468	LIC10714	Probable TonB-dependent receptor/outer membrane receptor protein
LB191	LIC20151	Putative TonB-dependent outer membrane receptor protein (Hbp A)
LA2510	LIC11458	Conserved hypothetical protein/outer membrane protein, porin superfamily
LA4337	LIC13479	Conserved hypothetical protein/PG-associated CM protein
LA0572	LIC12998	Conserved hypothetical protein/TonB-dependent outer membrane receptor
LA3258	LIC10881	Hypothetical protein/outer membrane protein, TonB dependent
LA2186	LIC11739	Conserved hypothetical protein

Lai locus: *L. interrogans *serovar Lai locus

Copen locus: *L. interrogans *serovar Copenhageni locus

When comparing the concordance or prediction agreement rates between the three prediction methods (excluding proteins with unknown localization by one or two programs), the rates for PSORTb and PA, PSORTb and ProtCompB, and PA and ProtCompB were 70.3%, 80%, and 59.5%, respectively. PSORTb was found to have a strong propensity to assign protein queries to CP and OM proteins, while PA was found to assign preferentially to CM, PP and EX proteins (p < 0.001, chi-square tests).

### Step 2: Homology-based and protein folding recognition predictions for non-consensus vote localizations

The non-consensus vote OM, EX, and PP proteins were further analyzed for localizations using DBsubloc, and GTD. As presented in Table 5, 6, 99 more proteins (43 out of 83 proteins predicted by two previous methods and 56 out of 617 proteins predicted by one previous method) were additionally identified as putative EX, while 48 proteins (23 out of 59 proteins predicted by two methods, and 25 from 980 proteins predicted by one method) were additionally identified as putative OM proteins as shown in Table 7, 8. Moreover, 58 proteins (20 out of 20 proteins predicted by two methods and 38 out of 504 proteins predicted by one method) were additionally predicted as PP proteins (Additional file 1). It is of interest that several protein loci currently annotated as hypothetical proteins without localization information were predicted in EX, OM and PP compartments by the combination method (Tables 3, 4, 5, 6, 7, 8, 9 and Additional file 1). The homology search and structural information from DBSubloc and GTD thus allowed further identification of EX, OM, and PP from the non-consensus vote set, however, 3725 protein localizations remain unknown.

**Table 5:** 43 Putative extracellular proteins (EX) derived from the 2 out of 3 predictions with significant DBSubloc or/and GTD prediction

**Lai Locus**	**Copen Locus**	**Protein annotation**	**SWISS-PROT^a^**	**PDB Code^b^**
LA1027	LIC12632	Sphingomyelinase C precursor (Sph1)/hemolysin	-	1bix
LA1029	LIC12631	Sphingomyelinase C precursor (Sph2)/hemolysin	-	1bix
LA4004	LIC13198	Sphingomyelinase C precursor hemolysin (Sph3)/sph- like	-	1bix
LA3540	LIC10657	Sphingomyelinase C precursor; hemolysin	-	1bix
LA3050	LIC11040	Hemolytic protein-like protein/hemolysin (sph4)	-	1aq0
LA3466	LIC10715	Thermolysin	P43133	1hyt
LA3454	LIC10723	Flagellar hook-associated protein(fliD)	Q9KWW7	1osp
LA3097	LIC11003	Treponemal membrane protein B precursor-like protein/LipL71	P19649	1l8w
LA1530	LIC12234	LRR containing protein	Q9RBS2	1d0b
LA1324	LIC12401	LRR containing protein/cytoplasmic membrane protein	-	1ogq
LA1354	LIC12375	LRR containing protein/cytoplasmic membrane protein	Q9RBS2	1ogq
LA2452	LIC11504	LRR containing protein/cytoplasmic membrane protein	Q9RBS2	1ogq
LA2862	LIC11180	LRR containing protein/cytoplasmic membrane protein	Q9RBS2	1ogq
LA2966	LIC11097	LRR containing protein/cytoplasmic membrane protein	Q9RBS2	1ogq
LA3324	LIC10831	LRR containing protein/conserved hypothetical protein	Q9RBS2	1ogq
LA3321	LIC10830	LRR containing protein/putative lipoprotein	Q9RBS2	1ogq
LA3322	LIC10830	LRR containing protein/putative lipoprotein	Q9RBS2	1ogq
LA0701	LIC12901	LRR containing protein/molybdate metabolism regulator	Q9RBS2	1ogq
LA2377	LIC11568	Peptidase, M23/M37/membrane associated peptidase	P24204	1acc
LA0505	LIC13050	Probable glycosyl hydrolase/conserved hypothetical protein	-	1f00
LA3725	LIC10502	Probable phenazine biosynthesis family protein/CM protein	-	1air
LA3730	LIC10498	Putative lipoprotein	P15921	1rmg
LA1368	LIC12364	Putative outer membrane protein/CagA	P47460	-
LA1759	LIC12050	Putative outer membrane protein/conserved hypothetical protein	Q52657	1czf
LA2443	LIC11507	Putative outer membrane protein/conserved hypothetical protein	Q9RBS2	1ogq
LA2447	LIC11505	Putative outer membrane protein/conserved hypothetical protein	Q9RBS2	1jl5
LA2450	LIC11505	Putative outer membrane protein/conserved hypothetical protein	Q9RBS2	1ogq
LA1915	LIC11990	TPR-repeat-containing proteins/cytoplasmic membrane protein	P80544	1qqe
LA0043	LIC10038	TPR-repeat-containing proteins/conserved hypothetical protein	Q9KQ40	1qqe
LA2773	LIC11246	Conserved hypothetical protein	Q06852	1l8w
LA3233	LIC10902	Conserved hypothetical protein	O83497	1qcx
LB001	LIC20001	Conserved hypothetical protein	-	1eut
LA1499	LIC12259	Conserved hypothetical protein/cytoplasmic membrane protein	P35825	1dab
LA1766	LIC12047	Conserved hypothetical protein/cytoplasmic membrane protein	Q07833	1czf
LA3333	LIC10825	Conserved hypothetical protein/cytoplasmic membrane protein	Q07833	1acc
LA2208	LIC11720	Conserved hypothetical protein/hypothetical protein	-	1e15
LA3276^c^	LIC10868	Conserved hypothetical protein/hypothetical protein	P15345	1dab
LA0022	LIC10021	Conserved hypothetical protein/putative lipoprotein	-	1dab
LA3210	LIC10920	Conserved hypothetical protein/putative lipoprotein	-	1rmg
LA3726	LIC10501	Conserved hypothetical protein/putative lipoprotein	Q9PJY2	1acc
LB216	LIC20172	Conserved hypothetical protein/putative lipoprotein	-	1wxr
LB225	LIC20176	Conserved hypothetical protein/putative lipoprotein	-	1wxr
LA4135^d^	LIC13296	hypothetical protein/putative lipoprotein	-	1koe

Note LRR: Leucine rich repeat

a: Swiss-Prot ID derived from DBsubloc database

b: PDB code derived from GTD prediction

c: Pfam: PF06739: SBBP (Seven Beta Blade Propeller domain)

d: pfam07588: DUF1554

**Table 6:** 56 Putative extracellular proteins (EX) derived from the 1 out of 3 predictions with significant DBSubloc or/and GTD prediction

**Lai Locus**	**Copen locus**	**Protein annotation**	**SWISS-PROT^a^**	**PDB code^b^**
LB258	LIC20197	Cysteine protease	-	1deu
LA0975	LIC12680	Fimh-like protein	-	1a6c
LA0858	LIC12930	Fimh-like protein/hypothetical protein	-	1dab
LA0492	LIC13060	LipL36 protein	-	1acc
LA3469	LIC10713	Iron-reglulated protein A/LruB/putative lipoprotein	-	1rmg
LA3075	LIC10464	Surface protein Lk90-like protein/Ig-like repeat domain	P35828	1dab
LA3778	LIC10464	Surface protein Lk90-like protein/Ig-like repeat domain	Q52657	1dbg
LA0378	LIC10325	TPR-repeat-containing proteins/hemolysin	Q98KC1	1a17
LA3138	LIC10973	Transmembrane outer membrane protein L1	-	1acc
LA1353	LIC12375	LRR containing protein	Q9RBS2	1jl5
LB196	LIC20154	LRR containing protein/lipoprotein	-	1d0b
LA0416^e^	LIC10365	Putative lipoprotein (LpL effector)	-	1gq8
LA0962^d^	LIC12690	Putative lipoprotein	-	1eut/1koe
LA1569^c^	LIC12208	Putative lipoprotein	P15345	1acc
LA2823^e^	LIC11207	Putative lipoprotein	-	1gq8
LA3064^e^	LIC11030	Putative lipoprotein	-	1czf
LA3848^c^	LIC13075	Putative lipoprotein	-	1qjv
LA3867	LIC13086	Putative lipoprotein	-	1cwv
LA1159	LIC12525	Putative outer membrane protein/putative lipoprotein	-	1cs6
LA1905	LIC11996	Putative outer rmembrane protein/hypothetical protein	-	1kit
LA1939	LIC11966	Putative outer membrane protein/hypothetical protein	-	1fio
LA2273	LIC11665	Putative outer membrane protein/hypothetical protein		1air
LA0563^d^	LIC13006	Hypothetical protein/putative lipoprotein (LenC)	-	1koe
LA0695^d^	LIC12906	Hypothetical protein/putative lipoprotein (LenA/LfhA/Lsa24)	-	1koe
LA1433^d^	LIC12315	Hypothetical protein/putative lipoprotein (LenD)	-	1koe
LA3103^d^	LIC10997	Hypothetical protein (LenB)	-	1koe
LA4073^d^	LIC13248	Hypothetical protein/putative lipoprotein (LenF)	-	1koe
LA4324^d^	LIC13467	Hypothetical protein/conserved hypothetical protein (LenE)	-	1koe
LA3370	LIC10793	Conserved hypothetical protein/surface antigen (Lp24)	-	1loq
LA0965	LIC12676	Conserved hypothetical protein	P25156	1d0b
LA1066	LIC12601	Conserved hypothetical protein	-	1dbg
LA1498	LIC12260	Conserved hypothetical protein	-	1ogq
LA2811	LIC11217	Conserved hypothetical protein	P25146	1ogq
LA3734	LIC10495	Conserved hypothetical protein/CM protein	-	1dab
LA3834^c^	LIC13066	Conserved hypothetical protein	P15345	1acc
LA4227	LIC13381	Conserved hypothetical protein	-	1sli
LA0663	LIC12930	Conserved hypothetical protein/hypothetical protein	-	1acc
LA0423^c^	LIC10371	Conserved hypothetical protein/putative lipoprotein	P15345	1qjv
LA1567^c^	LIC12209	Conserved hypothetical protein/putative lipoprotein	P15345	1czf
LA1568^c^	LIC12209	Conserved hypothetical protein/putative lipoprotein	P15345	1czf/1dbg
LA1691^c^	LIC12099	Conserved hypothetical protein/putative lipoprotein	-	1acc
LA3340^e^	LIC10821	Conserved hypothetical protein/putative lipoprotein	-	1ee6
LA3394^e^	LIC10774	Conserved hypothetical protein/putative lipoprotein	-	1gq8
LA3501	LIC10686	Conserved hypothetical protein/putative lipoprotein	-	1air
LA0283^c^	LIC10239	Hypothetical protein	-	1air
LA0426^c^	LIC10373	Hypothetical protein	P56964	1acc
LA0996^d^	LIC12668	Hypothetical protein	-	1koe
LA1764	LIC12048	Hypothetical protein	-	1qlg
LA1869	LIC12023	Hypothetical protein	-	1k14
LA2272	LIC11664	Hypothetical protein		1dab
LA3240	LIC10898	Hypothetical protein		1rmg
LA0074	LIC10067	Hypothetical protein/conserved hypothetical protein	-	1dbg
LA1065	LIC12602	Hypothetical protein/conserved hypothetical protein	-	1dab
LA1762	LIC12048	Hypothetical protein/conserved hypothetical protein	-	1qcx
LA3649	LIC10561	Hypothetical protein/conserved hypothetical protein	-	1qcx
LA3881	LIC13101	Hypothetical protein/OM with integrin like repeat domains	P35825	1dab

Note LRR: Leucine-rich repeat, a: Swiss-Prot ID derived from DBsubloc database, b: PDB code derived from GTD prediction, c: pfam06739: SBBP (seven bladed beta propeller) repeat d: pfam07588: DUF1554, e: pfam07602: DUF1565

**Table 7:** 23 Putative outer membrane proteins (OM) derived the 2 out of 3 predictions with significant DBSubloc or/and GTD prediction

**Lai locus**	**LIC locus**	**Protein annotation**	**SWISS-PROT^a^**	**PDB code^b^**
LA3471	LIC10711	Iron-reglulated protein A/cytoplasmic membrane protein	P12608	1i5p
LA1161	LIC12524	Long-chain fatty acid transport protein/fatty acid transport protein	-	1kmo
LA1100	LIC12575	Outer membrane efflux protein/cytoplasmic membrane protein	-	1ek9
LA1445	LIC12307	Outer membrane efflux protein/OM- TolC superfamily	P50468	1ek9
LA3685	LIC10537	Outer membrane protein/PG- associated periplasmic protein	P38369	1r1m
LA0056	LIC10050	Outer membrane protein OmpA family/PG-associated CM protein	Q05146	1r1m
LA2318	LIC11623	Predicted outer membrane protein/outer membrane protein	-	1a0t
LA1968	LIC11935	Putative outer membrane protein/conserved hypothetical protein	-	1a0t
LA2444	LIC11506	Putative outer membrane protein/outer membrane protein	-	1fep
LB110	LIC20087	Putative outer membrane protein/outer membrane protein	-	1uyn
LA2242	LIC11694	TonB-dependent outer membrane receptor	P46359	1fep
LA3242	LIC10896	TonB-dependent outer membrane receptor	P37409	1kmo
LA0465	LIC10405	TPR-repeat-containing proteins/conserved hypothetical	P58937	-
LA3675	LIC10544	Hypothetical protein/outer membrane protein	-	1a0t
LA2063	LIC11851	Conserved hypothetical protein/cytoplasmic membrane protein	-	1by5
LA3102	LIC10998	Conserved hypothetical protein	P76115	1nqe
LA3675	LIC10544	Hypothetical protein/outer membrane protein	-	1a0t
LA2168	-	Hypothetical protein	P43153	1a0t
LA3809	LIC10439	Hypothetical protein	-	1a0t
LA1501	LIC12258	Hypothetical protein	-	2mpr
LA3552	LIC10647	Hypothetical protein/conserved hypothetical protein	-	1kmo
LA2818	LIC11211	Hypothetical protein/conserved hypothetical protein	-	2mpr
LA4059	LIC13238	Hypothetical protein/conserved hypothetical protein	-	1by5
LB279	LIC20214	Hypothetical protein/conserved hypothetical protein	-	1kmo

Note a: Swiss-Prot ID derived from DBsubloc database, b: PDB code derived from GTD prediction

**Table 8:** 25 Putative outer membrane proteins (OM) derived from the 1 out of 3 predictions with significant DBSubloc and/or GTD prediction

**Lai locus**	**Copen Locus**	**Protein annotation**	**SWISS-PROT^a^**	**PDB Code^b^**
LA0616	LIC12966	LipL41/Outer membrane lipoprotein lipL41	-	1a17
LA2295	LIC11643	LipL45 protein	P02977	1l8w
LA0957	LIC12693	Outer membrane efflux protein/conserved hypothetical protein	P24145	1ek9
LA0581	LIC12990	Outer membrane efflux protein/conserved hypothetical protein	Q9ZHD2	1ek9
LA3733	LIC10496	Outer membrane efflux protein/conserved hypothetical protein	-	1ek9
LA0301	LIC10258	Outer membrane protein OmpA family/hypothetical protein	Q926C3	1r1m
LA0222	LIC10191	Outer membrane protein OmpA family/PG-associated CM protein	P22263	1r1m
LA1192	LIC12499	Putative outer membrane protein	-	1fep
LA1404	LIC12337	Putative outer membrane protein	-	2mpr
LA1931	LIC11975	Putative outer membrane protein/outer membrane protein	-	2mpr
LA1987	LIC11918	Putative outer membrane protein/conserved hypothetical protein	-	1osp
LB199	LIC20157	Putative outer membrane protein/conserved hypothetical protein	-	1fep
LA1030	LIC12630	TPR-repeat-containing proteins/hypothetical protein	P58937	1a17
LA0568	LIC13002	Conserved hypothetical protein	-	1kmo
LA1510	LIC12252	Conserved hypothetical protein	-	2mpr
LA0835	LIC12791	Hypothetical protein/conserved hypothetical protein	-	1fnf
LA2746	LIC11268	Hypothetical protein/conserved hypothetical protein	-	2mpr
LA2940	LIC11121	Hypothetical protein/conserved hypothetical protein	-	2mpr
LA2976	LIC11086	Hypothetical protein/conserved hypothetical protein	-	2mpr
LA3870	LIC13089	Hypothetical protein/conserved hypothetical protein	-	2mpr
LA4272	LIC13418	Hypothetical protein/conserved hypothetical protein	-	2mpr
LA4335	LIC13477	Hypothetical protein/conserved hypothetical protein	-	1kmo
LA0706	LIC12898	Unknown protein	P38370	1fep
LA1507	LIC12254	Unknown protein/outer membrane protein	-	1a0t
LA3853	LIC13078	Unknown protein/conserved hypothetical protein	-	1bxw

Note a: Swiss-Prot ID derived from DBsubloc database, b: PDB code derived from GTD prediction

**Table 9:** Protein subcellular localizations of *L. interrogans *predicted by PSORTb, PA, ProtCompB and the combination prediction

**Localization**	**Subcellular localization prediction**			
	
	**PSORTb**	**PA**	**ProtCompB**	**Combination prediction**
Cytoplasm (CP)	1125	921	2013	2690
Cytoplasmic membrane (CM)	606	715	1726*	813
Outer membrane (OM)	112	28		63
Periplasmic (PP)	30	86	478	75
Extracellular (EX)	29	326	510	114
Unknown	2825	2652	-	972

* Note that in ProtCompB prediction in this version, CM and OM were predicted as membrane proteins.

### Step 3: Cytoplasmic (CP) and cytoplasmic membrane proteins (CM) identified by Linear Discriminant Analysis (LDA)

The remaining 3725 proteins with unknown localization after step 2 were further analyzed using an LDA-based classifier we developed to identify CP and CM proteins using the set of CP and CM consensus outputs (418 CP proteins and 332 CM proteins) predicted by all of the three prediction programs (Additional file 2, 3) as a training set (see Materials and Methods). 2272 CP and 481 CM proteins were additionally identified from the 3725 "unknown set" by this approach (Additional file 4, 5). We also found that 66% (1501 out of 2272) of the LDA based predicted CP and 54% (260 out of 481) of the LDA based predicted CM are hypothetical or unknown proteins. In other words, overall 56.3 % (1516 out of 2690) of hypothetical and/or unknown proteins in the whole genome were assigned as CP and 38 % as CM or helix transmembrane proteins.

After the final step in the prediction method, we are able to confidently predict the localization of 3755 (79.4%) Leptospiral proteins. Our combination method thus has a considerably improved recall over the PSORTB and PA methods, approaching that of ProtCompB (Table 1). To test the final prediction accuracy with estimated % agreement and % coverage of our combination method, we then performed the localization prediction of 28 experimentally verified proteins from several studies of Leptospiral outer membrane and extracellular, or cell surface proteins.

### Protein subcellular localization prediction on the experimentally verified leptospiral outer membrane and extracellular proteins

As shown in the Additional file 6, the three prediction programs PSORTb, PA and ProtCompB gave markedly different predictions from one another for 28 experimentally OM and EX. Each of the three prediction programs had weaknesses, either poor agreement (ProtCompB) or low coverage (PSORTb and PA). Our combination approach was much better in the respect and showed good agreement and coverage.

## Discussion

Computational prediction for protein subcellular localization is a key step for genome annotation and development of drug and vaccine target. In this study, we used a combination method to putatively assign CP, CM, PP, OM, and EX proteins. We combined the results from three different algorithms namely PSORTb, PA and ProtCompB into a consensus vote to obtain higher prediction accuracy. The combination approach has previously been used to significantly reduce, or exclude false positive predictions for membrane topology prediction [33], and outer membrane prediction [34]. In our case, the accuracy of consensus vote is very high, since well characterized OM and EX proteins were predicted including lactonizing lipase [35], microbial collagenase [36], O-sialoglycoprotein endopeptidase [37], Rhs family protein [38], CsgA or C factor [39], thermolysin [40], leucine rich repeat proteins (LRR) [41-43], Ton-B dependent outer membrane receptor proteins, OmpA, porin, heavy metal efflux pump, TolC, and general secretory pathway protein D (Table 4).

On the other hand, the recall, or sensitivity of consensus vote prediction is low, especially for EX and OM. The recall for consensus vote is low, because PSORTb and PA programs are known to have limitations for some proteins. PSORTb requires a training set from a limited number of experimentally-determined proteins, while PA has a disadvantage in that query proteins have to share similarity to known proteins in the Swiss-Prot database [44]. Among high-throughput computational predictions for protein subcellular localization, PSORTb has been reported as the prediction tool that achieves the highest overall accuracy, followed closely by PA [22].

To overcome the limitations in PSORTb, PA and ProtCompB, the predictions for proteins predicted by only one or two out of the three prediction methods (the non consensus vote) were refined by homology-based search using the DBSubloc database and structural annotation in GTD. This allowed us to identify protein localizations with greater confidence. The advantage of GTD is that protein folding recognition or threading methods can determine pairs of proteins that have no obvious similarities in sequence, but have similar folds. It was previously suggested this approach should be carried out to increase prediction sensitivity for specific protein localization [22,45,46]. To our knowledge, this study is the first to employ GTD information to infer leptospiral protein localizations.

Structure-based information from GTD prediction revealed that the majority of the 99 EX predictions were proteins that may be secreted by the type III or the type V (autotransport) system. These proteins are shown in Table 5, 6 with their corresponding PDB code. Many of the putative EX proteins that are annotated as leucine rich repeat (LRR) containing proteins share sequence similarity to PopC protein (Q9RBS2), which is secreted through the hrp-secretion apparatus or the type III secretion pathway of *Ralstonia solanacearum *[41]. Structurally related well-characterized extracellular LRR proteins in other species include YopM (PDB code 1jl5), a *Yersinia pestis *cytotoxin [43], internalin B [47], a virulence factor of *Listeria monocytogenase *(PDB code 1d0b) and polygalacturonase inhibiting protein (PDB code 1ogq), a secreted protein involved in plant defense [48].

It is of interest to note that several *L. interrogans *proteins are contained within the LRR and TPR (Tetratricopeptide repeat) protein families, but predicted sub-cellular localization is not necessarily conserved among all members within each family (Table 3, 5, 6, 7, 8, 9 and Table in additional file 4). The majority of LRR proteins were predicted to be EX localized, while TPR proteins were predicted in all compartments except PP. This finding is consistent with the multiple functions of TPR homologues from more distantly related species in different sub-cellular milieux, including signal transduction, chaperone activity, cell-cycle, transcription, and protein transport [49,50].

Out of 48 non-consensus vote of predicted OM, 24 were proteins annotated as outer membrane or putative outer membrane proteins, while of the remainder were proteins annotated as conserved hypothetical proteins. The structural information derived from the GTD prediction of the conserved or hypothetical proteins that were predicted as putative OM were the same as that of the annotated outer membrane proteins. As shown in Table 7, 8, it can be observed that 24 hypothetical proteins can now be annotated as putative OM.

Although it is clear that the consensus vote combined with DB and GTD prediction can give robust prediction for EX, OM and PP, there are many proteins with either CP or CM localization remaining. Using our combination approach, we found that about 17% of genes encode putative CM proteins in *L. interrogans *serovar Lai genome, which is of similar proportion to the 20% – 30% CM proteins in other bacterial species [25,51]. From our subcellular location prediction we identified 63 OM and 114 EX proteins as potential vaccine candidates. On the other hand, it is possible to exclude 813 CM and 75 PP predicted proteins as vaccine candidates, on the basis of their localization.

We compared our predictions with the previously published works. We found that 10 of 16 membrane proteins predicted by Gamberini *et al*. 2006, including four also demonstrated to be immunogenic among 8 pathogenic serovars in that study, were also predicted by our method as membrane proteins (2 EX, 1OM, 1PP and 6 CM) [18]. We examined the localizations of the 145 putative lipoproteins reported by Setubal *et al*. [19], and found 29 EX, 2 OM, 7 PP and 26 CM proteins among 125 probable lipoproteins, and 1 PP and 3 CM among 21 possible lipoproteins. The localizations of 63 putative lipoproteins could not be identified, which included proteins containing signal peptidase II recognition sites and proteins lacking sequence and/or structural homology to known membrane proteins (see Additional file 7). Spirochaetal lipoproteins are found in four subcellular compartments: the periplasmic leaflet of the cytoplasmic membrane, the periplasmic outer leaflet of the outer membrane, or beyond the outer membrane into the environment as extracellular proteins [52]. Therefore, 15 of the 145 putative lipoproteins identified as CP by our method are unlikely to be lipoproteins because of their localization. These false positive lipoproteins include UDP-glucose 6-dehydrogenase, cell-division protein, regulator of chromosome condensation RCC1 family, and 3-oxoacyl- [acyl-carrier protein] reductase. The frequency of falsely-identified lipoproteins just exceeds the reported 1% false positive rate for the SpLip program [52]. Our results can be considered as complementary to those reported by Setubal *et al*. [52], and increase the accuracy of lipoprotein prediction.

We also compared our predictions with the 226 leptospiral surface exposed protein predictions (extracellular, outer membrane, periplasmic, inner (cytoplasmic) membrane by their localization definition) reported by Yang *et al*. [20] and found a concordance of 38.5 % (87/226) (see Additional file 8). We think the discrepancies arise from false assignments generated by the prediction algorithms used, which can be identified by comparison with proteins for which there are reliable experimental data of localization (see Additional file 6) [2-14,53-57]. Our predictions have a higher coverage and agreement with the experimentally tested *L. interrogans *protein set than the study by Yang *et al*. [20], suggesting that our prediction method may be of greater overall utility for genome annotation of membrane proteins. After manual inspection of predicted localizations, we found further examples of possible false assignments. The greatest discrepancy was found for 42 proteins were identified as CM by our method, but OM by Yang *et al*. Some proteins among this group have homologues in other species for which there is experimental evidence of CM location, including methyl-accepting chemotaxis protein mcpB [58], aerotaxis sensor receptor [59], and penicillin-binding protein [60].

It was found that several loci without localization annotation were assigned by the combination prediction method. Therefore, we propose that the annotations with respect to subcellular localization for these loci can be tentatively revised. Among this group of proteins, we noted additional similarities to known protein families. One prominent group with the the SBBP domain (seven beta blade propeller proteins, Pfam PF06739) contain 9 hypothetical proteins: LA0283 (LIC10239), LA0423 (LIC10371), LA0426 (LIC10373), LA1567 (LIC12209), LA1568(12209), LA1569 (LIC12208), LA1691 (LIC12099), LA3276 (LIC10868), LA3834 (LIC13066). Three loci annotated as hypothetical proteins or lipoproteins, namely LA0996 (LIC12668), LA0962 (LIC12690), and LIC13296 (LA4135), were predicted as EX localized (shown in Table 5, 6), and may belong to the Len (leptospiral endostatin-like lipoproteins) family, based on conservation of DUF1554 domain (pfam PF07588) and structural similarity to mammalian endostatin-like protein (PDB 1koe). These proteins act as adhesion proteins and bind to host extracellular matrix (ECM) [53,57] or human factor H [56]. (Table 5, 6 and Table in the Additional file 6). Furthermore, three loci LIC11207 (LA2823), LIC10821 (LA3340) and LIC10774 (LA3394) and LIC10365 (LA0416), previously described to have similarity with the leptospiral effector protein [54] were identified as putative EX proteins in agreement with their proposed immunomodulator function.

Our combination prediction method has high agreement and coverage of experimentally verified OM and EX proteins (see Additional file 6). On the other hand, experimental localization studies are limited by insufficient sensitivity to detect low abundance proteins and cross contamination of cellular compartments during sample purification, as discussed previously by Rey *et al*. [21]. It is of note that several predicted PP proteins in this work e.g. FlaB1 periplasmic flagellin (LA2017/LIC11890) have previously been identified as possible PP contaminants in experimental studies of OMV proteins [13,20]; hence our prediction method may help in correct interpretation of future experimental verification studies, thus leading to better predictions in uncharacterized genomes. However, it should be emphasized that no automatic prediction can be accurate without experimental verification.

## Conclusion

In this study, we have demonstrated that the specificity and sensitivity of protein subcellular localization prediction can be improved by incorporation of multiple predictive methods and structural information. By this approach, localizations can be assigned to previously hypothetical *L. interrogans *proteins. We think this approach is applicable for subcellular localization predictions in other prokaryote proteomes, with the caveat that some predictions are robust than others, i.e. CP and CM better than OM, EX or PP.

## Materials and Methods

### Data sets

Amino acid sequence queries were 4,727 proteins of *Leptospria interrograns *serovar Lai genome (chromosome I: NC_004342, chromosome II: NC_004343) [15] and 3,728 protein ORFs of *Leptospira interrogans *serovar Copenhageni strain (Fiocuz L1-I30) [accession number AEO16823 (chromosome I) and AEO16824 (chromosome II) [17] obtained from GenBank. Two datasets of proteins with known subcellular localization were used. One was an experimentally confirmed data set containing 278 CP and 309 CM of Gram-negative bacteria described by Gardy *et al*. 2003 [28] and used for validation of the LDA based classifier's performance. Another one was a 299 protein-data set containing 145 CP, 69 CM proteins, 29 PP, 38 OM and 18 EX which was the testing data previously used to evaluate various protein localization predictions in Gardy and Brinkman [22].

### Computational Data sets mputational prediction tools for *in silico *protein localization

Several publicly available programs were used in combination of predictions. Protein subcellular localization for Gram-negative bacteria was carried out using PSORTb [27,28], Proteome analysis (PA) [29], and ProtCompB [30]. Feature based predictions for signal peptide sequence and α helix transmembrane proteins were identified using SignalP [23] and TMHMM [24,25] respectively.

### Homology based searching and structural annotation

Homology search for subcellular localization information was carried out using BLAST search against DBSubloc, a localization specific protein database [31]. A protein folding recognition method for structural information used to predict the fold of protein sequence with distant homology to known structure was performed using homology search against GTD (the Genomic Threading Database) [32].

### Prediction strategy (as shown in Figure 1)

#### Step 1. Consensus votes prediction

We reasoned that more accurate protein subcellular localization predictions can be gained from the consensus of methods. All leptospiral protein queries were analyzed using three subcellular localization prediction tools for Gram-negative bacteria, namely PSORTb, Proteome analysis (PA), and ProtCompB for cytoplasm (CP), cytoplasmic membrane (CM), periplasmic (PP), outer membrane (OM) and extracellular proteins (EX). Note that in this version ProtCompB prediction, CM and OM are not distinguished so both proteins are predicted as membrane proteins. The consensus prediction for each sequence was calculated using a simple majority vote type procedure. If all 3 methods agree for localization, it is assigned as a "consensus vote". The remaining results (1 or 2 out of 3 predicted) were assigned as "non-consensus vote". The CP and CM proteins assigned in this step were used as a training set for the development of LDA based classifier for CP and CM in a the next step.

#### Step 2. Homology-based and protein folding recognition prediction

Homology based and structural information can also be used to infer the potential localization site of query proteins [22,45,46]. Therefore, the remaining query proteins assigned as non-consensus vote results of PP, OM and EX were further analyzed for sequence and structure homology. Since subcellular localization is an evolutionarily conserved trait, if a protein query is homologous to a known protein with the same localization, the localization was assigned. The protein query sequences were compared to proteins in DBSubloc database at *E*-value ≤ 10^-3 ^using BLAST search. Structure annotation of these queries was also performed using GTD prediction. The query proteins sequences were assigned to structures (shown as PDB code) with the high level of probability prediction (certain and high) for these protein queries. In this study, the confidence range based on p-value of measuring the reliability of the structure annotation as certain (0 ≤ p < 0.01%) and high (0.01% ≤ p < 0.1%) were considered as a statistically significant structure annotation.

#### Step 3. Identification of putative CP and CM using the LDA based classifier

A number of putative CP and CM identified as non-consensus vote results was further analyzed by SignalP and TMHMM. The feature attributors derived from SignalP and TMHMM predictions were then integrated and analyzed using the LDA based classifier. Proteins classified with probabilities ≥ 0.9 to be CP or CM proteins were taken as significant. The remaining queries that could not be identified in this step were classified as "unknown" results.

### LDA based Classifier for CP and CM

We developed a specific classifier using the training set driven from the consensus vote prediction of leptospiral CP and CM proteins to increase the accuracy of prediction. In the classification-based prediction, our classifier was built on an LDA algorithm analyzing the value of multiple character vectors of SignalP-NN, SignalP-HMM and TMHMM prediction results of the set of training sequences. The accuracy of the LDA based classifier was investigated using leave-one out cross validation. We used experimentally determined or known CP and CM proteins of Gram-negative bacteria previously performed in the evaluation of PSORTb as a test dataset for validation of the LDA based classifier's performance [27]. Overall, the accuracy of LDA based classifier achieved 94.96%.

## Authors' contributions

WV and SI participated in designed the research project. SI and EP carried out the computational analysis and developed LDA-based classifier. WV analyzed and interpreted the result, drafted and produced the manuscript. PP provided the further insights for refining the manuscript. All authors read and approved the final manuscript.

## Supplementary Material

Additional file 1 **Putative PP proteins in *L. interrogans *serovar Lai genome**. This table lists the Lai locus and protein annotation of (A) 17 predicted PP derived from the consensus vote prediction (B) 20 predicted PP derived from 2 out of 3 predictions with significant DBsubloc and/or GTD predictions, (C) 38 predicted PP derived from 1 out of 3 predictions with significant DBsubloc and/or GTD predictions. Click here for file

Additional file 2 **Putative CP proteins predicted by the consensus vote prediction in *L. interrogans *serovar Lai genome**. This table lists the Lai locus and protein annotation of 418 predicted CP proteins derived from consensus vote and used as the training set for the development of the LDA based classifier. Click here for file

Additional file 3 **Putative CM proteins predicted by the consensus vote prediction in *L. interrogans *serovar Lai genome**. This table lists the Lai locus and protein annotation of 332 predicted CM proteins derived from consensus vote and used as the training set for the development of the LDA based classifier. Click here for file

Additional file 4 **Putative CP proteins predicted by LDA based classifier of *L. interrogans *serovar Lai genome**. This table lists the Lai locus and protein annotation of 2272 predicted CP proteins predicted by LDA based classifier Click here for file

Additional file 5 **Putative CM proteins predicted by LDA based classifier of *L. interrogans *serovar Lai genome**. This table lists the Lai locus and protein annotation of 481 predicted CM proteins predicted by LDA based classifier. Click here for file

Additional file 6 **Subcellular localizations of 28 experimentally studied OM and EX proteins of *L. interrogans *serovar Lai**. This table lists the protein name, *L. interrogans *serovar Lai and copenhengeni locus, experimental localization, subcellular localization prediction using PSORTb, ProtCompB, PA, and the combination prediction of 28 experimentally studied OM and EX proteins. Click here for file

Additional file 7 **The result of subcellular localization of putative lipoproteins using the combination method**. This table lists the Lai locus tag and protein annotation of 125 probable lipoproteins and 21 possible lipoproteins predicted by SpLip programs [19] and the subcellular localization of these lipoproteins predicted by the combination method. Click here for file

Additional file 8 **Subcellular localization of vaccine candidate using the combination method.**. This table lists the Lai locus tag and protein annotation of 226 vaccine candidate predicted by Yang *et al*. [20] and the subcellular localization of these vaccine candidates predicted by the combination method. Click here for file
