# Genome-wide subcellular localization of putative outer membrane and extracellular proteins in *Leptospira interrogansserovar* Lai genome using bioinformatics approaches

**DOI:** 10.1186/1471-2164-9-602

**Published:** 2008-12-16

**Authors:** Wasna Viratyosin, Supawadee Ingsriswang, Eakasit Pacharawongsakda, Prasit Palittapongarnpim

**Affiliations:** 1 BIOTEC Central Research Unit, National Center for Genetic Engineering and Biotechnology, Pathumthani, 12120, Thailand; 2 Department of Microbiology, Faculty of Science, Mahidol University, Bangkok, 10400, Thailand

## Correction

After the publication of this work [1], we discovered a typographic error in Table 6 (56 Putative extracellular proteins (EX) derived from the 1 out of 3 predictions with significant DBSubloc or/and GTD prediction). For the entry of LA3370/LIC10793 (a conserved hypothetical protein/surface antigen) [1], the homologous structure predicted by GTD should read, "1l0q", a structural surface layer of protein binding containing tandem beta propeller. In the final print version, it appeared as 1loq (letter o replacing zero). This distinction is important as 1loq is the PDB structural code for a different protein, Orotidine 5'-phosphate decarboxylase, which is localized as cytoplasmic and not extracellular or secreted.
